# The Elusive Origin of Glioblastoma: Where Do We Stand?

**DOI:** 10.3390/cells15070590

**Published:** 2026-03-26

**Authors:** Monica Pernia Marin, Hamed Almabrok, Michael L. Miller, Aya Haggiagi

**Affiliations:** 1 Division of Neuro-Oncology, Department of Neurology, Columbia University Vagelos College of Physicians and Surgeons, New York, NY 10032, USA; mp4424@cumc.columbia.edu; 2 New York-Presbyterian Hospital, Columbia University Irving Medical Center, 710 W 168th Street, 9th Floor, New York, NY 10032, USA; 3 Department of Medicine, Saint Michael’s Medical Center, Newark, NJ 07102, USA; 4 Department of Pathology and Cell Biology, Columbia University Vagelos College of Physicians and Surgeons, New York, NY 10032, USA; 5 Herbert Irving Comprehensive Cancer Center, New York, NY 10032, USA

**Keywords:** glioblastoma, cell of origin, neural stem cells, oligodendrocyte precursor cells, RNA sequencing, spatial transcriptomics, multi-omics, glioma models, bioinformatics, artificial intelligence

## Abstract

Glioblastoma (GBM) remains one of the most lethal cancers, and despite advancements in understanding its underlying molecular signature, effective therapeutics are still lacking. The multifaceted challenges of designing treatments for GBM are compounded by the inability to identify a definitive cell of origin, the understanding of which is crucial for developing impactful therapies and ultimately improving patient outcomes. High-resolution technologies, including single-cell and single-nucleus RNA sequencing, spatial transcriptomics, multi-omics, next generation glioma models, bioinformatics, and artificial intelligence are creating an important opportunity to comprehensively map the cellular origin of GBM and its evolutionary dynamics. Accumulating evidence support neural stem cells (NSCs) and oligodendrocyte precursor cells (OPCs) as primary candidates, providing critical insights into the ontogeny of GBM. This comprehensive review synthesizes current knowledge on the cellular origins of GBM and evaluates advanced methodologies, deepening our understanding of its development.

## 1. Background

### 1.1. Incidence

Glioblastoma (GBM) represents approximately 50% of all malignant primary brain tumors [1]. Incidence correlates positively with age, peaking in the eighth decade of life [1]. Prognosis remains poor with a median overall survival (OS) of 15 months [2]. Outcomes scale inversely with age [3], and survival is significantly shorter in older populations, particularly octogenarians, who have a median OS of 2 to 3.5 months [3,4]. Current therapeutic failures stem from a complex interplay of molecular and biological drivers of invasion and treatment resistance. Central to these challenges is the “elusive” nature of the GBM cellular origin. In this context, “GBM cell of origin” refers to the cellular population that undergoes initial malignant transformation and should not be confused with the stem-like tumor cells (glioma stem cells or GSCs) observed within established tumors. GSCs are a result of tumor evolution, but they are not the precursor cells that initiate the disease [5].

### 1.2. Cell of Origin Influence on Molecular Pathogenesis

This tumor is characterized by profound intertumoral and intratumoral heterogeneity as a consequence of the accumulation of genetic alterations, dysregulated signaling, and developmental traits inherent to the cell of origin [6,7]. Neural stem cells (NSCs), oligodendrocyte precursor cells (OPCs), and even mature glial cells are established candidates for gliomagenesis [7,8]. Critical progenitor functions, including maintenance, proliferation, and differentiation, are regulated by multiple signaling pathways that are frequently altered in GBM, including phosphoinositide 3-kinase (PI3K)/protein kinase B (Akt)/mammalian target of rapamycin (mTOR) and rat sarcoma (RAS)/rapid accelerated fibrosarcoma(RAF)/mitogen-activated protein kinase (MAPK) [9,10,11,12].

PI3K/Akt/mTOR signaling maintains the NSC/progenitor pool via activation by epidermal growth factor (EGF) and fibroblast growth factor 2 (FGF2). While independent inactivation of PI3K or mTOR reduces the proliferation of neural/progenitor cells without altering stemness, simultaneous inhibition induces astrocytic differentiation and exit from the progenitor state [12]. Vitucci et al. showed that concurrent activation of PI3K and MAPK through oncogenic Kirsten rat sarcoma virus oncogene homolog (*KRAS*) activation and/or phosphatase and tensin homolog deleted on chromosome ten (*PTEN*) loss drives proliferation, migration, and invasion in G1/S checkpoint-deficient murine astrocytes. These cells also adopted transcriptional signals that mirror the proneural GBM subtype in humans [13].

Alterations in epidermal grow factor receptor (EGFR) occur in approximately 40–50% of GBMs [14,15,16], whereas alterations in platelet-derived growth factor receptor alpha (PDGFRA) occur in ~10–20% of cases [17], making EGFR the most frequently altered receptor tyrosine kinase in this tumor [15,16]. In neurodevelopment, EGFR regulates normal NSC expansion, differentiation, migration, and self-renewal [18,19]. PDGFRA is required for oligodendrogenesis from NSCs in the subventricular zone (SVZ). Jackson et al. observed that excessive PDGF activation in the SVZ induces hyperplasia with distinct glioma features. Thus, EGFR and PDGFR signaling are primary determinants of NSCs and OPCs biology, and their activation predetermines tumor’s transcriptional states that are associated with the cell of origin and persist in advanced disease [20].

Canonical Wnt/β-catenin signaling promotes the self-renewal and proliferation of glioma stem cells (GSCs), facilitating tumor growth and treatment resistance [21,22,23]. Systematic analysis of 65 publications showed that Wnt, Janus kinase/signal transducer and activator of transcription (JAK/STAT3), PI3K/Akt, and Notch pathways help maintain stemness, lineage transitions, and coordinate interactions with the microenvironment [22,24]. Liu S et al. examined the interaction between regulatory T cells (Tregs) and GSCs and found that Tregs promote stemness via the transforming growth factor-beta (TGF-β)/nuclear factor kappa-light-chain-enhancer of activated B cells (NF-κB)/interleukin 6 (IL-6)/STAT3 signaling [25].

Cell cycle dysregulation, primarily through alterations in the retinoblastoma protein (RB) and the tumor protein 53 (TP53) pathways, also permits unchecked expansion while cells preserve a progenitor-like state [26,27,28]. The RB protein pathway has been found to be altered in 78.9% of GBMs, mostly through cyclin-dependent kinase inhibitor 2A (*CDKN2A*) deletion followed by amplification of cyclin-dependent kinase 4 and 6 (*CDK 4/6*), or by direct *RB1* mutation/deletion (7.6%). Concurrently, the TP53 pathway has been found to be dysregulated in 85.3% of GBMs through several genetic alterations, including the mutation or deletion of the *TP53* gene in ~28% of cases [16]. Hongwu et al. showed that co-deletion of *TP53* and *PTEN* in the CNS of mice triggers acute-onset high-grade gliomas (HGGs), similar to primary GBM in humans [28].

Telomerase reverse transcriptase (*TERT*) promoter mutations occur in 66–83% of GBMs and have diagnostic and prognostic value with the shortest OS observed in patients with concomitant *EGFR* amplification [29,30]. These mutations perpetuate mechanisms that maintain telomere length and cell immortalization [31]. Miki et al. explored the impact of heterozygous *TERT* promoter mutations in neuronal precursor cells derived from human-induced pluripotent stem cells (hiPSCs). Results showed that the *TERT* C2287 mutation led to growth advantages after telomere shortening in neural progenitors in vivo [32].

These molecular pathways illustrate that cells of origin—while not sufficient to initiate oncogenesis on its own—influence tumor behavior and therapeutic vulnerability throughout the disease course.

### 1.3. Current Management and Therapeutic Resistance

Current treatment protocols do not yet incorporate cellular ontogeny into clinical decision-making, not due to lack of biological relevance, but because the fundamental understanding of GBM’s cellular origin remains insufficient to confirm lineage-specific drivers or translate these findings into clinical action. Management is generally guided by neuroanatomical location, patient performance status, and a narrow set of established molecular biomarkers. Surgical resection is the primary and most impactful intervention. A 2023 report from the international Response Assessment in Neuro-oncology (RANO) resect group demonstrated that maximal safe and near-total resection significantly improved outcomes compared to submaximal resection or biopsy alone [33]. Improved survival was also observed in patients who had supramaximal resection, defined as the removal of both contrast-enhancing and non-contrast-enhancing tumor volumes [33]. Following surgery, current National Comprehensive Cancer Network^®^ (NCCN^®^) guidelines recommend standard fractionated radiation therapy (RT) delivered in 60 Gy fractions over approximately 6 weeks with concurrent temozolomide (TMZ), followed by adjuvant TMZ combined and alternating treatment fields (TTF; Category 1) [34].

Among molecular biomarkers, O-6-methylguanine-DNA methyltransferase (*MGMT*) promoter methylation remains the most clinically informative, given its established predictive and prognostic value. Tumors harboring *MGMT* promoter methylation often exhibit increased sensitivity to alkylating agents and improved clinical outcomes [35,36]. Treatment decisions are further stratified by patient age and Karnofsky performance status (KPS). In patients over 70 years of age with favorable KPS (≥60) and methylated or indeterminate *MGMT* promoter status, hypofractionated RT is recommended (category 1). Conversely, older patients with poor KPS (<60) are typically treated with hypofractionated RT, TMZ monotherapy, or best supportive care [37].

Enrollment in clinical trials is the preferred option for patients with progressive GBM when available [34]. Outside of trial settings, salvage therapies include re-resection, re-irradiation (category 2B), and TTF (category 2B). Systemic options consist primarily of alkylating agents, including TMZ re-challenge, lomustine (CCNU), and carmustine (BCNU). With further disease progression or treatment intolerance, etoposide (category 2B) or platinum-based agents (category 3) may be considered [34]. Bevacizumab, a monoclonal antibody targeting vascular endothelial growth factor (VEGF), is primarily used to mitigate vasogenic edema and radiation necrosis. While this treatment serves as an effective steroid-sparing agent and improves progression-free survival (PFS), it has not demonstrated an OS benefit in large-scale trials [34,38]. Regorafenib is an oral multikinase inhibitor that showed OS benefit in a Phase II trial of patients with recurrent GBM compared with lomustine (7.4 months versus 5.6, respectively) (REGOMA trial) [39]. These results could not be confirmed in a subsequent Phase III clinical trial (GBM AGILE) and regorafenib’s accrual was stopped for futility [40]. Although the repertoire of clinically “actionable” alterations in GBM remains limited, the molecular targets listed in Table 1 are increasingly relevant in the setting of disease progression or for when there is intolerance to other available treatments [34].

The standardized approaches are frequently met with therapeutic resistance, a consequence of transcriptional landscapes defined by cellular lineage and adaptive changes acquired over time [7,41]. GSCs are more resistant to TMZ than the bulk of tumor cells because they possess more robust DNA repair mechanisms such as elevated MGMT activity and radiation-sensitive 51 protein (RAD51) driven pathways [42,43]. In addition, GCS can limit the apoptotic activity of TMZ, which favors a therapy-induced senescent state that facilitates survival and recurrence [42,44]. RT can also induce a senescent state in GSCs. These senescent cells are not biologically inert; they can persist after treatment, maintain stemness, secrete pro-tumorigenic senescence-associated secretory phenotype (SASP) factors, and later re-enter the cell cycle to promote tumor progression [45].

Overall, resistance is compounded by aberrant signaling cascades, epithelial-to-mesenchymal transition (EMT), and the persistence of GSCs [46]. Additional barriers include restricted drug delivery across the blood–brain barrier (BBB) and an immunosuppressive microenvironment that favors tumor growth [47,48]. Consistent with these challenges, numerous clinical trials have failed to yield meaningful survival benefits, even for agents with preclinical rationale or efficacy in other malignancies with overlapping molecular features [49,50,51,52,53,54,55,56,57,58,59,60,61,62] (Table 2). Immune checkpoint inhibitors exemplify this disconnect; despite transformative success in melanoma and lung cancer, these therapies have repeatedly failed to show significant benefit in GBM [57,58,59,60,61,63]. A recent Phase II trial combining pembrolizumab with re-irradiation in patients with recurrent GBM showed improved OS only in patients previously treated with bevacizumab, with no benefit observed in bevacizumab-naïve patients [64]. Similarly, *EGFR*-targeted therapies (including those targeting amplification) have faltered due to the activation of compensatory molecular pathways, inadequate CNS penetration, and treatment-limiting toxicity [65].

The persistent lack of effective durable responses across diverse therapeutic strategies suggests that even mutation-informed, personalized treatments may fail to account for the biological heterogeneity stemming from disparate developmental origins. Defining how cellular ontogeny shapes therapeutic sensitivity and lineage-specific resistance will be critical for advancing biologically informed therapies, a necessary step in the management of this formidable cancer.

**Table 1:** NCCN-recommended targeted therapies and corresponding mutations in GBM.

Target Mutation	Agent (s)	Considerations
***NTRK*** **gene fusion**	Larotrectinib Entrectinib Repotrectinib	Repotrectinib (NCCN^®^ category 2B) [34]
***FGFR*** **alterations**	Erdafitinib	Primarily targets *FGFR2* and *FGFR3* fusions; NCCN^®^ Category 2B [34]
***BRAF*** **V600E**	BRAF/MEK inhibitors: dabrafenib/trametinib vemurafenib/cobimetinib	Highest prevalence in epithelioid GBM [66]

The National Comprehensive Cancer Network^®^ (NCCN^®^) [34], GBM: glioblastoma, *NTRK*: neurotrophic tyrosine receptor kinase, *FGFR*: fibroblast growth factor receptor, *BRAF*: v-Raf murine sarcoma viral oncogene homolog B, *MEK*: mitogen-activated protein kinase.

**Table 2:** Main negative clinical trials in GBM.

Therapeutic Class	Trial/Study	Setting	Agent (s)	Main Outcomes
**Antiangiogenic therapy**	AVAglio [49]	Newly diagnosed	Bev + Standard therapy	Improved PFS without OS benefit
	RTOG 0825 [50]	Newly diagnosed	Bev + Standard therapy	No improvement in OS.
	BELOB [51]	Recurrent	Bev ± CCNU	No significant OS benefit
	EORTC 26101 [52]	Recurrent	Bev + CCNU vs. CCNU alone	PFS benefit without OS improvement.
	TAMIGA [53]	Recurrent	Bev continuation	No survival advantage
	Cediranib (Phase III) [54]	Recurrent	Cediranib ± CCNU	No survival advantage
	Enzastaurin (Phase III) [55]	Recurrent	Enzastaurin vs. CCNU	No survival advantage
	Cilengitide (CENTRIC) [56]	Newly diagnosed	Cilengitide + Standard therapy	No improvement in OS.
**Immune checkpoint inhibition**	CheckMate 143 [57]	Recurrent	Nivolumab vs. Bev	No OS benefit
	CheckMate 498 [58]	Newly diagnosed (MGMT-unmethylated)	Nivolumab + RT	No survival improvement
	CheckMate 548 [59]	Newly diagnosed (MGMT-unmethylated)	Nivolumab + RT/TMZ	No survival benefit
	Ipi-Glio [60]	Newly diagnosed	Ipilimumab + TMZ	No improvement in PFS or OS
	Pembrolizumab (Phase II) [61]	Recurrent	Pembrolizumab ± Bev	Failed to improve survival
**Protein kinase inhibitors**	Systematic Review (Da Silva et al.) [62] Meta-analysis of RCTs (Pinto-Fraga et al.) [67]	Newly diagnosed/recurrent	*EGFR*, *PI3K*, *AKT*, *mTOR* inhibitors	No significant benefit in OS or PFS
	Trial by CIGNO [68]	Recurrent	Gefitinib	No meaningful clinical benefit
**Cytotoxic chemotherapy**	*BrUOG329* [69]	Recurrent	Irinotecan + TMZ	No OS benefit
	Field et al. (Phase II study) [70]		Carboplatin + Bev vs. Bev alone	No survival improvement
	Reardon et al. (Phase II study) [71]		Carboplatin + Irinotecan + Bev	Modest activity
**Monoclonal antibodies**	Westphal et al. (Phase III study) [72]	Newly diagnosed	Nimotuzumab (EGFR antibody) + Standard therapy	Failed to demonstrate efficacy
**Alkylating chemotherapy**	Systematic review and meta-analysis (Gupta et al.) [73]	Newly diagnosed	Extended TMZ	Apparent survival benefit of extended TMZ was mostly derived nonrandomized comparative studies.
	Norden et al. (Phase II study) [74]	Recurrent	Dose-intensified TMZ	Marginal efficacy

RCTs: randomized controlled trials, Bev: bevacizumab, CCNU: lomustine, TMZ: temozolomide, RT: radiation therapy, OS: overall survival, PFS: progression-free survival, MGMT-methylated/unmethylated: O-6-methylguanine-DNA methyltransferase (*MGMT*) promoter methylation status, *EGFR*: epidermal growth factor receptor, *PI3K*: phosphatidylinositol 3-kinase, *AKT*: serine/threonine kinase (also known as protein kinase B), *mTOR*: mammalian target of rapamycin, EORTC: European Organisation for Research and Treatment of Cancer, CIGNO: Gruppo Italiano Cooperativo di Neuro-Oncologia.

## 2. The Complex Landscape of GBM Cellular Origins

A fundamental challenge in neuro-oncology centers on the identification of GBM’s cell of origin. Owing to this challenge, the definition has evolved over the last several decades. Initially thought of as reflecting a specific, static cell state in which driver oncogenic mutations first achieve dominance immediately before tumorigenesis, it is now thought to reflect dynamic lineage-competent populations that possess the specific epigenetic and regulatory flexibility to harbor, and eventually initiate, a malignant state. The complex GBM biology is characterized by pronounced cellular heterogeneity and transcriptional plasticity, with cells frequently occupying molecular states that mirror distinct progenitor-like hierarchies, complicating efforts to infer lineage of origin based on tumor phenotype or transcriptional identity alone [41,75]. Furthermore, oncogenic transformation itself can activate quiescent developmental programs or induce dedifferentiation, confounding the identity of the initiating cell [76,77].

**Figure 1:**
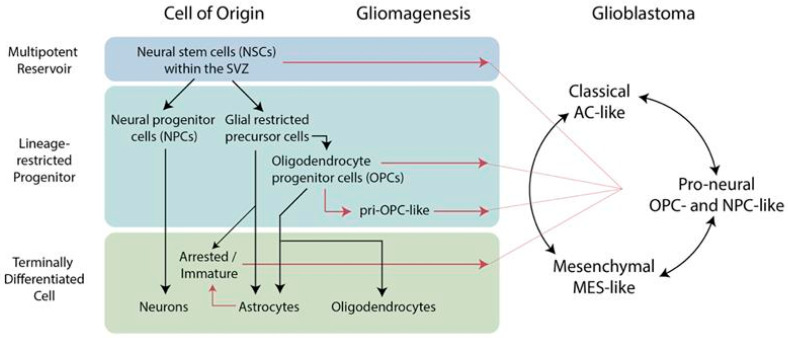
Developmental trajectory and potential cellular origins of glioblastoma (GBM). Gliomagenesis may occur in multipotent reservoirs of neural stem cells (NSCs) or lineage-restricted progenitors, such as oligodendrocyte progenitor cells (OPCs). Additionally, gliomagenesis may occur in terminally differentiated astrocytes via a process of dedifferentiation (or reversion), or may occur in immature astrocytic precursors nearing terminal differentiation. Black arrows indicate physiologic trajectory while red arrows indicate potential routes of gliomagenesis.

Recent developments in high-resolution and multi-layered profiling have introduced a critical dimension to GBM research, moving beyond the limitations of traditional bulk tissue analysis. Within this dimension, it is possible to interrogate how lineage competence, genetic context, and cellular plasticity within and across specific anatomical niches integrate to allow malignant transformation, addressing “the who, the where, and the how” of GBM ontogenesis. NSCs and OPCs have stood as the most strongly supported candidates for gliomagenesis, while astrocytes and other differentiated glial cells may serve as cells of origin following oncogenic dedifferentiation under certain genetic and microenvironmental conditions (Figure 1). The following discussion examines the biological and experimental evidence for each of these cell populations with a particular focus on how the integration of advanced methodologies is refining our understanding of their role in GBM development.

### 2.1. Neural Stem Cells (NSCs)

The multipotent NSCs within neurogenic niches in the adult brain, particularly those residing in the SVZ, have long been considered a leading candidate for the cellular origin of GBM. Experimental animal models show that introducing glioma-relevant driver genetic alterations into NSCs is sufficient to initiate glioma development. These findings suggested that NSCs possess the lineage competence required to sustain gliomagenesis [78], and were further supported by complementary studies showing the lineage continuity of GBM cells to NSCs within the SVZ by tracing *TP53*-mutant GBM cells’ expression patterns [79]. Consistent with an early progenitor model to malignant transformation, Alcantara Llaguno et al. genetically modified cells by introducing specific oncogenic alterations across successive stages of neural lineage, demonstrating that glioma-initiating potential is highest in NSCs and declines sharply with neural differentiation [80].

In human GBM, deep sequencing mirrored this observation by showing direct evidence of concurrent oncogenic mutations between tumor tissue and NSCs within the SVZ. Lee et al. utilized deep targeted sequencing of GBM-associated genes, *TERT* promoter amplicon sequencing, and single-cell sequencing to analyze matched GBM tissue, normal SVZ, and cortex/blood from 28 patients. The study identified shared somatic driver mutations, most notably within the TERT promoter, between tumor tissue and histologically normal-appearing cells within the SVZ in 97% of patients [81]. A subsequent genomic study by the same group tested triple-matched specimens of an expanded cohort of 60 patients. The study confirmed recurrent genetic overlaps between tumor-free SVZ tissue and matched tumors, validating prior findings [82]. In addition, it showed that patients with these shared SVZ-tumor mutations had shorter PFS and OS, suggesting that the specific oncogenic events in the SVZ may play a role in tumor-aggressive biology.

NSCs appear to also impact tumor phenotypes. Jacques et al. examined whether genetic alterations introduced specifically within the adult NSC compartment in the lateral wall of the SVZ could influence tumor phenotype. The study assessed the effects of distinct combinations of tumor suppressor gene alteration on tumor development and found that disruption of different genetic pathways within adult NSCs led to the development of tumors with different histopathological features; however, the same alterations did not yield similar phenotypes when introduced outside of the NSC compartment [83].

Beyond tumor initiation, cells in the SVZ niche are increasingly implicated in GBM progression and post-treatment disease persistence. Despite not being a study specific to SVZ, whole-exome sequencing of 69 multi-region samples (tumor core, infiltrative margins, and the SVZ) from 11 patients with GBM were analyzed using phylogenomic reconstruction. The results showed that early infiltrative subclones present in residual disease compartments, including the SVZ (6/11 patients), persisted after treatment and contributed to recurrent tumor growth in longitudinally sampled cases [84]. The same group more recently characterized the cellular landscape of the SVZ niche. Deep sequencing and copy number analysis in combination with single-nucleus RNA sequencing (sn-RNA seq) and spatial transcriptomic profiling were used to assess the genetic and cellular composition of matched samples from original tumor mass and distant SVZ. These samples were collected from 15 patients at the time of surgery. The study identified tumor cells within the SVZ that exhibited a transcriptional “blueprint” distinct from those in the main tumor mass, including enrichment of mesenchymal-associated gene expression centered on zinc finger E-box binding homeobox 1 (*ZEB1*). It also demonstrated that the SVZ niche was enriched with tumor-associated microglia and macrophages with compartment-specific signaling interactions [85]. While this study is not specific to NSCs, nor did it analyze longitudinal recurrences, it does provide evidence of the potential critical role of cells within the SVZ niche to sustain a malignant state, especially when further contextualized by the findings of Li et al. who directly examined whether SVZ-resident NSCs contribute to GBM reconstruction following surgical resection. Using lineage tracing and transcriptomic analyses, the study revealed that NSC populations within the SVZ carrying tumor-associated mutations survived primary therapy and gave rise to recurrent tumor populations [86].

### 2.2. Oligodendrocyte Precursor Cells (OPCs)

One of the persistent questions in GBM ontogeny is whether it arises from a multipotent NSC in a “reservoir” or from lineage-restricted progenitor cells like OPCs, and whether these progenitor cells represent an independent origin or a required downstream stage for mutation-harboring NSCs. OPCs are distributed throughout the adult brain parenchyma and retain proliferative capacity and lineage plasticity beyond developmental periods, characteristics that confer a potential susceptibility to oncogenic reprogramming. Assanah et al. first demonstrated this independent sufficiency by showing that retroviral delivery of PDGF into glial progenitor cells in adult rat white matter induces rapid and consistent formation of tumors with histologic features of GBM. These progenitor cells of origin have oligodendrocyte lineage [87]. This finding was further supported in genetically engineered mouse models (GEMMs) where controlled deletion of *TP53* and *NF1,* specifically in adult OPCs, was sufficient to initiate malignant gliomas. This study also showed that even quiescent adult progenitors possess the innate lineage competence required for transformation, and that the transformation process is a dynamic multistep process rather than a continuous expansion. It includes an initial transient hyperproliferative spike, followed by a period of relative dormancy, during which mutant OPCs showed impaired differentiation, and finally, a malignant conversion phase. The transcriptomic profiling of these OPC-derived tumors most closely resembled the proneural subtype of human GBM and retained lineage-associated gene expression features [88].

Lineage tracing investigations further complicate this hierarchical relationship with NSCs. Lie et al. demonstrated that even when mutations originate in NSCs, it is exclusively the OPC progeny that undergoes the aberrant expansion prior to tumor formation. Using a genetic mosaic system known as Mosaic Analysis with Double Markers (MADM), concurrent mutations in *TP53* and *NF1* were initiated sporadically in NSCs, and the progeny of these mutant cells were tracked through lineage labeling. Although initial alterations were introduced in NSCs, significant expansion prior to malignancy was observed only in OPCs and not in other NSC-derived lineages or even the NSCs themselves. When the same *TP53* and *NF1* mutations were introduced directly into OPCs, gliomas formed, and resulting tumors showed signatures consistent with OPC origin [89]. An intriguing thought based on this result is that the oligodendrocyte lineage may act as a “bottleneck” for malignant transformation, regardless of where the initial oncogenic driver alteration occurs. The prevalence of this lineage program in human GBM was identified by Neftel et al. [41]. The study utilized sc-RNA seq and found that malignant cells occupy four cardinal transcriptional states with different distributions in each tumor, one of which is OPC-like. The OPC-like state was characterized by the enrichment of canonical oligodendrocyte precursor cell markers including PDGFRA, oligodendrocyte transcription factor 1 (OLIG1), oligodendrocyte transcription factor 2 (OLIG2), and SRY-box transcription factor 10 (SOX10). This transcriptional signature does not directly identify OPCs as the originating cell within the established tumor, but it demonstrates that a significant subset of malignant cells retains the signature of an oligodendrocyte-lineage ancestor [41].

A more recent critical work by Kim et al. sought to further characterize this interplay by providing a high-resolution map of the evolutionary trajectory of GBM from niche to tumor. Using a spontaneous somatic mouse glioma model that permits the accumulation of oncogenic alterations prior to overt tumor formation enabled the identification of a distinct population of precancerous OPC-like (pri-OPC-like) cells before radiographic or histologic evidence of tumor. These cells represent an intermediate state characterized by the early acquisition of oligodendrocyte lineage programs such as OLIG2 and SOX10 expression prior to development of large-scale genomic imbalances. To assess relevance in human disease, the study included histologically tumor-free SVZ tissue from GBM patients which were sampled at distant anatomical locations from the primary tumor mass. Single-cell transcriptomic profiling of human SVZ revealed cell populations transcriptionally similar to the precancerous states observed in the mouse model. The study also showed that these precancerous cells undergo discrete transcriptionally defined stages of acquiring oncogenic features as gliomagenesis progressed, including increased proliferative signaling, alterations in extracellular matrix organization, and activation of survival and metabolic programs. In later stages, cells derived from the pri-OPC-like population displayed expanded oncogenic signatures and increased transcriptional diversity, contributing to intratumoral heterogeneity in established GBM [90].

### 2.3. Differentiated Glial Cells

A challenge to linear progenitor models of GBM ontogeny is the evidence that terminally differentiated glial cells, including mature astrocytes and possibly oligodendrocytes, can also serve as cells of origin through their potential for oncogenic dedifferentiation. This capacity was demonstrated in mature astrocytes using GEMMs where loss of tumor suppressors *TP53* and *NF1* triggered the formation of HGGs that are indistinguishable from NSC-derived tumors [77]. A critical study by Sojka et al. recently showed that astrocyte susceptibility to transformation is neither uniform nor ubiquitous, but instead depends on developmental state and regional identity. High-resolution mapping of human astrocyte maturation trajectories revealed that GBM transcriptional programs frequently align with intermediate or “middle-stage” astrocytic states, rather than fully mature populations [91]. This finding implies that GBM may arise from astrocytes that are developmentally arrested or have reverted to an immature regulatory configuration. Supporting this view, recent multi-omics analyses have shown that astrocytes may be uniquely predisposed to oncogenesis when specific genetic alterations coupled with microenvironmental stress allow them to bypass the limitations of their terminal differentiation [92].

The oncogenic potential may extend to other differentiated cell populations, possibly through similar mechanisms of lineage “reversion”. Sher et al. recently demonstrated that GBMs initiated from mature oligodendrocytes display distinct transcriptional features enriched for myelin-related gene expression and are associated with lineage-specific neurological phenotypes characterized by early-onset motor deficits in vivo [93]. However, it is important to note that the evidence to support mature oligodendrocytes as a GBM cell of origin is more restricted.

## 3. Technological Advances in GBM Cellular Origins Research

**Figure 2:**
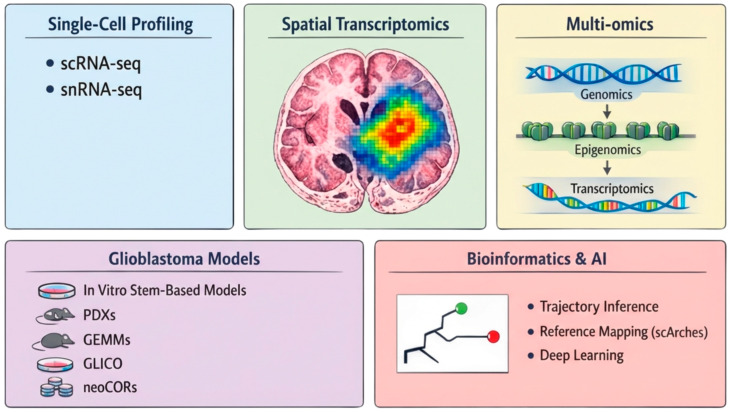
Multimodal experimental and computational approaches used in GBM cellular origins. Figure Legend: Advanced technologies, including single-cell and single-nucleus RNA sequencing (sc/snRNA-seq), provide high-resolution data necessary to reconstruct developmental hierarchies and identify plausible cells of origin. Spatial transcriptomics preserves anatomical context by mapping gene expression patterns onto histologically defined brain regions such as the subventricular zone (SVZ). The multilayers of multi-omics can link mature tumor cells back to their ancestral lineages. Patient-derived insights are validated through a range of models including in vitro stem-based systems, genetically engineered mouse models (GEMMs), patient-derived xenografts (PDXs), GBM cerebral organoid model (GLICO), and neoplastic cerebral organoids (neoCORs). Computational methods such as trajectory inference, deep learning, and reference mapping using single-cell architectural surgery (scArches) are then applied to align these diverse datasets to effectively map GBM transcriptional states onto neurodevelopmental and disease-specific atlases.

High-resolution and multidimensional methodologies are providing the granularity necessary to investigate the transient cellular states and lineage relationships underlying gliomagenesis. Population-averaged genomic analyses established the broad molecular landscape of GBM; however, they lacked the resolution necessary to interrogate tumor-initiating states. Single-cell/single-nucleus, spatial, and integrative multi-omics now permit a detailed mapping of cellular populations across distinct anatomical niches. Coupled with improved glioma models and more sophisticated computational tools, these methodologies are building additional dimension(s) for a more critical delineation of the early events preceding overt GBM formation (Figure 2).

### 3.1. Single Cell/Single Nucleus RNA Sequencing

scRNA-seq and snRNA-seq have become essential methods in evaluating cellular hierarchies of gliomagenesis. scRNA-seq enables the transcriptomic profiling of viable individual cells, providing the resolution necessary to segregate neoplastic and non-neoplastic populations and identify transcriptionally distinct tumor subpopulations within GBM [94]. However, enzymatic dissociation of dense brain tissue introduces technical inaccuracies as selective loss or stress-induced transcriptional alterations in fragile, large, or highly differentiated cells can skew cellular representation [95]. snRNA-seq profiles nuclear RNA isolated from frozen or archival tissue and serves as a complementary, often preferred method for human GBM specimens. By minimizing dissociation-related limitations, snRNA-seq allows a more uniform sampling of diverse tumor and microenvironmental cell populations and permits the analysis of archival biobank cohorts with established clinical histories [96,97].

Application of these methods has revealed that GBM cells occupy a spectrum of transcriptional states that partially resemble programs associated with normal neural lineages, including NSC, OPC, and astrocytic-like states [41,94]. These signatures provide the evidentiary basis for implicating NSCs or lineage-restricted progenitors as cells of origin, but they also identify a significant degree of oncogenic reprograming and the resulting “flexible” lineage-associated states, with cells exhibiting substantial transcriptional plasticity transitioning between states in response to genetic alterations, microenvironmental signals, or therapeutic pressure [76,98]. Because this inherent fluidity can obscure the ancestral lineage, integrating single-cell profiling with additional molecular and spatial information is required to more comprehensively contextualize the relationship between GBM origin and the resulting tumor structure.

### 3.2. Spatial Transcriptomics and Cellular Mapping

Spatial transcriptomics provides the anatomical context often lost in dissociated single-cell studies. Whereas dissociative approaches define cellular states and lineage-associated identities, spatially resolved methods retain information about “where” these programs are organized within the tumor and the surrounding brain. This spatial organization is essential for understanding GBM origin, as the developmental lineage programs and microenvironmental signals that influence malignant initiation are inherently structured in space [76].

These technologies broadly comprise two methodological categories: sequencing-based and imaging-based approaches. Sequencing-based methods utilize the principles introduced by Ståhl et al. in which tissue sections are placed onto arrays containing spatially barcoded capture oligonucleotides that allow transcriptome-wide RNA profiling while preserving positional information [99]. Bead-based extensions of this concept, such as Slide-seq and Slide-seqV2, increase spatial resolution by using densely packed, barcoded beads to reconstruct gene expression patterns at near single-cell scale following computational decoding [100,101]. More recent implementations of array-based capture systems have substantially increased resolution by employing continuous or near-continuous capture surfaces, reducing the spatial gaps inherent in earlier designs, resulting in a more precise assignment of transcripts to individual cells. Imaging-based spatial transcriptomic approaches, including multiplexed error-robust fluorescence in situ hybridization (MERFISH) and sequential fluorescence in situ hybridization (seqFISH+), bypass the need for capture arrays by directly visualizing RNA molecules within intact tissue sections through iterative rounds of probe hybridization and imaging [102,103]. These methods enable the detection of hundreds to thousands of predefined transcripts at subcellular resolution while preserving cellular morphology and spatial relationships. Newer versions of these technologies have expanded their multiplexing capacity, allowing for the simultaneous detection of RNA transcripts and protein markers within the same tissue section. This multi-modal capability is particularly valuable in GBM, where key aspects of neoplastic and immune cell identity are defined by protein markers that may not be fully captured at the transcriptomic level.

Utilizing spatial methods in GBM cellular origin research has overcome the “geographic” disconnect between ancestral reservoirs and distant masses which has historically confounded lineage tracing. These platforms, for example, permit the identification of latent oncogenic clones, supporting a link between anatomical niche and tumor initiation [81]. They also delineate how the signaling architecture of a specific niche might “license” the transition of a quiescent ancestral cell into a lineage-committed malignant progenitor [90].

### 3.3. Multi-Omics

Integrative multi-omics combines transcriptomic data with genomic alterations, epigenomic features (such as chromatin accessibility and histone modifications), and at times proteomic or metabolic information to capture the multi-layered regulatory basis that dictates how tumor cell identity is established during initiation [104]. For GBM cellular origin, this data could potentially permit the identification of epigenetic memory, where a cell’s chromatin landscape remains “primed” with its ancestral cellular origin despite oncogenic reprogramming of its transcriptome. As a result, it allows for the high-resolution distinction between inherited lineage traits and states acquired through plasticity or dedifferentiation [105,106].

The previously discussed study by Kim et al. utilized a single-cell assay for transposase-accessible chromatin (scATAC-seq) and snRNA-seq to reconstruct the transition from a latent ancestral reservoir to a malignant progenitor. By mapping the chromatin accessibility landscape of histologically normal SVZ cells, the study identified a distinct “precancerous” regulatory state characterized by the early opening of loci associated with the pri-OPC like signatures. This epigenetic priming occurs prior to overt transcriptional changes and could provide the answer to the mechanistic “how” for how specific genetic mutations are able to execute a malignant transformation within a defined lineage [90].

Although not cellular origin-specific, Liu et al.’s study also highlighted the importance of multi-omics in investigating GBM, combining scATAC-eq and snRNA-seq to investigate transcriptional and chromatin accessibility differences between cells located in the center of the tumor and those infiltrating brain tissue [107]. GBM cells that invaded surrounding brain tissue had gene expression patterns that were more similar to OPCs than to astrocytes.

### 3.4. Advanced Glioma Models

Developing models for GBM research has been difficult because intratumoral conditions and the numerous interactions between tumor cells and the surrounding microenvironment are hard to reproduce in the laboratory.

#### 3.4.1. In Vitro Stem-Based GBM Models

An early work by Lee et al. demonstrated that tumor stem cells (TSCs), maintained under NSC conditions, formed multicellular spheres (“neurospheres”) and preserved the genetic, transcriptional, and biological characteristics of the original tumors. In contrast, cells grown in standard serum-containing media progressively deviated from their parental tumors. This work showed that glioma stem-like cells are a more reliable system to study GBM biology [108]. Then, Hubert et al. developed a three-dimensional GBM organoid culture system from patient-derived GSCs that reproduced the stem cell heterogeneity seen in vivo [109]. Because it replicated intratumoral hypoxic gradients, it was also possible to investigate interactions between hypoxic and non-hypoxic stem cell populations [109].

#### 3.4.2. Patient-Derived Xenografts (PDXs)

PDXs are generated by engrafting freshly resected patient tumor tissue into immunodeficient mice. Orthotopic implantation preserves tumor architecture and mirrors its growth and molecular features. They can be used to study tumor cell interactions in the native brain microenvironment and for drug testing [110]. Yet, they have their limitations, as do the other models discussed here. Ben-David et al. showed that PDXs undergo rapid mice-specific genomic changes during passaging. This means that genetic features that are clinically relevant in humans may be lost in PDXs over time, which limits investigations on cell of origin, late tumor progression, and therapeutic response [111].

#### 3.4.3. Genetically Engineered Mouse Models (GEMMs)D

These models allow tumors to arise de novo within an intact CNS and immune system through the introduction of oncogenic mutations into NSCs, astrocytes, OPCs or other defined neural lineages. Some examples include mice with a complete loss of *TP53* and *PTEN*, combined with RTK signaling pathway activation (e.g., PDGF or EGFR). These alterations originate GBM-like tumors with distinct phenotypes depending on the targeted cell population [112,113]. Since these models are based in mice, they are genetically less complex and different between species, which are important limitations. Even so, they are valuable to study how the cell of origin influences GBM identity, plasticity, and progression [112,113].

#### 3.4.4. Syngeneic Murine Glioma Models

Syngeneic models, such as GL261, CT-2A, and SMA-560, are created by the implantation of murine glioma cells into immunocompetent mice of the same genetic upbringings. The use of these models is recommended to study interactions between the tumor and the immune system, as well as with therapy response [114].

#### 3.4.5. Cerebral Organoid-Based Models

Ogawa et al. showed that genetically engineered human cerebral organoids can generate GBM-like tumors with invasive properties and in vivo tumorigenicity [115]. Then, Linkous et al. developed the GBM cerebral organoid model (GLICO) by integrating patient-derived GSCs with human embryonic stem cell (hESC)-derived cerebral organoids [116]. In this model, GSCs migrated into the organoid tissue where they proliferated, infiltrated, formed tumor microtubes, and organized into tumors that closely resembled patient GBMs [116].

#### 3.4.6. Engineered and Bioprinted Organoid Systems

Bioprinted GBM organoids combine patient-derived tumor cells with a decellularized porcine brain extracellular matrix or “bioink” with a printed layer of human endothelial cells. This model recapitulates GBM invasive behavior, hypoxic gradients, and cellular heterogeneity, but they are resource intensive and lack normal brain tissue to explore physiologic tumor–brain interactions [117]. Neoplastic cerebral organoids (neoCORs) are a 3D in vitro model developed by Bian et al. through the introduction of oncogenic mutations into cerebral organoids. This research group recommend using neoCORs to study tumor invasiveness and other features of tumor biology, as well as to test drug effects in presence of certain genetic alterations [118]. A limitation is that this model does not capture the full genomic complexity of advanced GBM [118,119,120].

#### 3.4.7. Lessons from Comparing Organoid Models

Organoid models are designed to represent both the tumor and its neural environment, but there are meaningful variations across models [119]. Pine et al. described tumor cell subpopulations in four different GBM models, including two-dimensional glioma spheres (2D), three-dimensional tumor organoids (TO), GLICO, and PDXs [121]. GLICO showed the strongest transcriptional similarity to the original patient tumors and most effectively recapitulated the full spectrum of GSCs states and key GBM gene programs found in primary tumors [121]. Most experts in this area state that choosing the right model is a “judgement call” that depends on the experimental question since organoid systems have different but complementary properties.

### 3.5. Bioinformatics and Artificial Intelligence (AI)

Analytical tools in bioinformatics permit the investigation of the GBM cell of origin across high-dimensional datasets. Rather than replacing experimental studies, these methods provide a systematic evaluation of the cellular signatures accessible during the earliest stages of tumor formation [122].

A primary strength of bioinformatics is multimodal integration, merging a wide range of heterogenous biological, spatial, and clinical information to evaluate underlying tumor evolution and heterogeneity. An earlier study by Corces et al. is an important example of multimodal integration in oncology. Analyzing massive data from chromatin accessibility (ATAC-seq), whole-genome sequencing, transcriptomics, and clinical phenotypes across thousands of samples from human cancers revealed that tumor transcriptional states are not random; rather, they remain tethered to the developmental lineage of the cell of origin. This suggests that oncogenic mutations do not simply “override” developmental constraints, but instead operate preferentially within chromatin contexts that are already permissive to specific malignant transitions [123].

Trajectory reconstruction methods such as pseudotime analysis and RNA velocity are now widely applied to GBM datasets to map directional relationships among tumor cell transcriptional states [124,125]. They allow the distinction of early, lineage-compatible states from later adaptive or therapy-induced states by modeling cells’ movements through transcriptional space. When tumor cells retain gene expression signatures resembling NSCs, OPCs, or differentiated glial lineages, trajectory analyses can indicate plausible cells of origin; however, it is important to note that this is based on transcriptional similarity rather than direct lineage tracing and, therefore, does not prove ancestry [124,126].

Machine learning provides the analytical power needed to recognize patterns across large datasets that exceed the capacity of manual interpretation. Supervised and unsupervised learning approaches are now used to classify cells based on their similarity to established reference atlases of human and murine development. This was demonstrated in the Neftel et al. study, which classified GBM cells based on their similarity to reference atlases of brain development, helping isolate the identities that persist through oncogenesis [41]. An important recent development in this area is the work by Lotfollahi et al., who introduced an updated deep learning method called single-cell architectural surgery (scArches), using transfer learning to directly map new independent single-cell datasets onto existing high-resolution reference atlases. This approach allowed for the minimization of model training complexity and increased efficiency of analysis while mitigating technical noise frequently found between datasets. There are certainly multiple applications to this advanced precise projection of data, including the study of tumor initiation and drug development [127].

Now, with AI, there is an even greater capacity for predictive modeling with deep generative models, which are being increasingly optimized and tested in the different aspects of oncology, from early diagnosis to monitoring [128]. Their use in cell of origin identification is particularly relevant given their potential ability to bridge molecular identity with anatomical context and help distinguish intrinsic lineage features from environmentally induced states [129].

## 4. Novel Therapeutic Modalities

Therapeutic approaches that directly target the cell of origin are not yet available. This reflects both the incomplete definition of GBM cellular origins and the lack of definitive lineage-specific drivers that can be translated into clinical interventions. As a result, current investigational modalities do not attempt to selectively eliminate defined initiating populations, but instead exploit biological properties associated with candidate cells of origin, such as tumor tropism and niche engagement, to improve drug delivery, enhance tumor selectivity, or amplify antitumor immune responses.

### 4.1. Stem Cell-Mediated Delivery

The therapeutic application of stem cells in GBM is predicated based on the evidence discussed in previous sections supporting NSCs and other progenitor populations as plausible cells of origin. In preclinical models, NSCs selectively migrated toward tumor foci, potentially as a consequence of the same lineage programs responsible for oncogenesis, tumor invasion, and resistance in GBM. This intrinsic tropism for the glioma microenvironment could provide a mechanism to circumvent the selective permeability of the blood–brain barrier (BBB), positioning stem cells as versatile vehicles for targeted drug delivery [130,131,132].

Bagó et al. developed tumor-derived induced NSCs (iNSCs) engineered to secrete a variant of the tumor necrosis factor α-related apoptosis-inducing ligand (TRAIL; iNSC-sTR), which is a pro-apoptotic ligand [133]. When co-cultured with GBM cells, iNSCs retained their capacity to proliferate and differentiate. Again, during in vitro migration assays, iNSCs selectively migrated toward GBM cells. When implanted in the frontal lobe of mice, viable iNSCs were detected for at least a month post-implantation, demonstrating that these cells can survive in vivo. Another important finding in this study was that iNSC-sTR cells maintained TRAIL expression without losing their stem cell properties and had tumoricidal activity with improved survival in tumor-bearing mice [133].

Metz et al. expanded this concept by engineering NSCs to express carboxylesterases (CEs). These cells were able to locally convert irinotecan (CPT-11) into its active metabolite (SN-38) within the tumor bed, enhancing tumor sensitivity while reducing systemic toxicity [134]. A later study by Portnow et al. reported the first in-human intracerebral delivery of NSCs engineered to express cytosine deaminase (CD), which converts 5-fluorocytosine (5-FC) to 5-fluorouracil (5-FU). This study included patients with recurrent HGG, confirmed CD-NSC migration to the tumor sites, and local drug activation [130]. A subsequent dose-escalation study by the same group established the feasibility and safety of this approach and provided a recommended dose for Phase II evaluation [135]. Ongoing trials are also combining NSCs with oncolytic viruses to maximize viral delivery and intratumoral spread [136,137]. In more common terms, these studies seek to materialize the idea of stems cells being the “Trojan Horse” against GBM.

Gunnarsson et al. explored how NSCs can be used to modulate the immune response. They observed a higher density of intratumoral T-cells in rats treated with a combination of NSC-delivered IL-7 and peripheral immunization with interferon gamma (IFN-γ)-transduced autologous tumor cells, which ultimately caused tumor regression [138]. These findings suggest that NSCs could potentially augment antitumoral T-cell response and counteract immune evasion by tumoral cells [132,138].

### 4.2. Oncoviruses

Adenovirus and other oncolytic viruses such as herpesvirus, reovirus, and measles are being evaluated in clinical trials for GBM, either as monotherapies or in combination with established treatments [132,139,140,141]. This idea is appealing because oncolytic viruses can exert antitumor effects through complementary mechanisms, including direct tumor cell lysis with subsequent release of tumor-associated antigens, induction of apoptosis, and activation of both adaptive and innate antitumor immune responses [142]. As mentioned in our prior section, NSCs are being evaluated as vectors for the delivery of oncolytic viruses in GBM. A study by Allen et al. showed that the measles virus had a cytopathic effect on GSCs in vitro. In subsequent experiments, the investigators infected GSCs with measles strains before these were implanted in the right caudate nucleus of mice. Their results showed significantly prolonged survival in the treated mice compared to control (*p* = 0.0483). Lastly, using two different GSCs xenograft models (GBM6 and GBM12) the investigators compared the effect of active (MV-GFP) and inactivated measles strains. They found that MV-GFP-treated animals had a longer survival when compared with inactivated virus-treated controls, and these differences were statistically significant in both xenograft models (GBM6 *p* = 0.0021, GBM12 *p* = 0.0416) [143].

### 4.3. Senolytic Agents

As mentioned earlier, TMZ and RT can precipitate a senescent state in GSCs. The resilience of these cells exploits the inherent durability of normal neural stem and progenitor cells, suggesting that strong survival programs are inherited from the cells of origin. Preclinical studies have begun to explore whether senolytics can eliminate this persistent population of cells. Beltzig et al. conducted in vitro experiments testing the senolytic compounds ABT-737 and ABT-263 (navitoclax) in senescent GBM cells that had been previously treated with TMZ. Both agents demonstrated senolytic activity through the inhibition of B-cell lymphoma (Bcl2) anti-apoptotic family proteins [144]. The investigators also treated GBM senescent cells with irradiation or lomustine and found that neither of them had senolytic activity, which could partially explain tumors’ resistance to these therapies in the clinical setting [144]. Senolytic therapies cannot yet be recommended clinically, but the preclinical evidence is encouraging since these agents target the senescent stem-like cells most responsible for tumor regrowth.

## 5. Conclusions

The cellular origin of GBM remains unresolved, but current evidence most strongly implicates NSCs and OPCs as lineage-competent candidates with differentiated glial populations capable of malignant transformation under certain genetic and microenvironmental conditions. With the continued expansion of multidimensional technologies, including single cell profiling, spatial transcriptomics, multi-omics, glioma models, bioinformatics, and AI, the field is well positioned to accelerate the resolution of existing knowledge gaps in GBM initiation and evolution. Although therapies that directly target initiating cell populations are not yet available, emerging strategies that leverage lineage-associated properties, such as cell–cell-mediated delivery systems and oncolytic viral approaches, illustrate how improved resolution of cellular origins may ultimately inform more precise interventions and improve patient outcomes.

## Data Availability

No new data were created or analyzed in this study.
